# Challenges of palliative care in children with inborn metabolic diseases

**DOI:** 10.1186/s13023-018-0868-5

**Published:** 2018-07-09

**Authors:** Jessica I. Hoell, Jens Warfsmann, Felix Distelmaier, Arndt Borkhardt, Gisela Janßen, Michaela Kuhlen

**Affiliations:** 1 Medical Faculty, Department of Paediatric Oncology, Haematology and Clinical Immunology, Centre for Child and Adolescent Health, University of Duesseldorf, Moorenstr. 5, 40225 Duesseldorf, Germany; 2 Medical Faculty, Department of General Paediatrics, Neonatology and Paediatric Cardiology, Centre for Child and Adolescent Health, University of Duesseldorf, Moorenstr. 5, 40225 Duesseldorf, Germany

**Keywords:** End-of-life care, Children, Metabolic disease, neurological disease, Palliative care

## Abstract

**Background:**

Our objective was to evaluate children with metabolic diseases in paediatric palliative home care (PPC) and the process of decision-making. This study was conducted as single-centre retrospective cohort study of patients in the care of a large specialized PPC team.

**Results:**

Between 01/2013 and 09/2016, 198 children, adolescents and young adults were in the care of our PPC team. Twenty-nine (14.6%) of these patients had metabolic conditions. Median age at referral was 2.6 years (0–24), median duration of care 352 days (3–2248) and median number of home visits 13 (1–80). Most patients are still alive (16; 55.2%). Median number of drugs administered was 5 (range 0–12), antiepileptics were given most frequently.

Symptom burden was high in all children with metabolic disorders at referral and remained high throughout care. Predominant symptoms were gastrointestinal, respiratory and neurologic symptoms.

Children with metabolic conditions, who were referred to PPC younger than 1 year of age had a shorter period of care and died earlier compared to those children, who were referred to PPC later in their lives (older than 10 years of age).

Eleven (37.9%) of the children initially had no resuscitation restrictions and 7 (53.8%) of those who died, did so on ICU.

**Conclusions:**

About 15% of children with life-limiting conditions in PPC present with metabolic diseases. Symptom burden is high with neurologic, respiratory and gastrointestinal symptoms being the most frequent and most of those being difficult to treat. In these children, particular attention needs to be addressed to advance care planning.

**Electronic supplementary material:**

The online version of this article (10.1186/s13023-018-0868-5) contains supplementary material, which is available to authorized users.

## Background

The number of children, adolescents and young adults with life-limiting conditions (LLCs) and, thus, a right of paediatric palliative care (PPC) is increasing [1]. However, most data on symptoms and needs of children in PPC refer to those with cancer, whereas the majority of the children is living with or dying from LLCs other than cancer [2]. Little is known on the symptoms and needs of these children.

The most common diagnoses in PPC are genetic and congenital conditions including metabolic diseases and most of the children present with some degree of cognitive impairment [3]. In the subgroup of children with metabolic diseases such as mitochondrial disorders, peroxisomal diseases or certain lysosomal storage diseases, for whom curative treatment is not possible, almost all present with severe neurological impairment and are nonverbal. Due to a progressive loss of inhibitory control of the CNS, most affected children face a broad range of symptoms [4]. Treatment intends to modify disease trajectory and to obtain best possible symptom control and care. However, the underlying symptom pathology often remains enigmatic impeding causative symptom control.

Phases of disease stability change with times of acute deterioration. Often, such acute episodes come along with incomplete recovery and the children remain on a reduced new health plateau. In addition, the families encounter the uncertainty of not knowing which episode will become life-threatening. Beyond acute health exacerbations, likewise the disease trajectory remains highly uncertain, with an overall decline of health, which often occurs slowly over months to years [5].

Caring for a child with incurable metabolic disease and neurological impairment and, thus, impaired communication, confronts caregivers and treating physicians with three substantial problems: The difficulty to correctly identify distressing symptoms, to accurately determine the cause of the symptom and to sufficiently treat it as most of those are highly intractable [6, 7]. In addition, due to the wide range and severity of presenting problems, various subspecialists need to be involved in PPC.

Thus, the overall aim of the study was to (first) determine the number and diagnoses of affected children in this patient group and (second) to analyse whether care needs differ in this presumably heterogeneous group of diagnoses from patient to patient.

## Methods

This study was conducted as single centre analysis of patients referred to a specialised paediatric palliative care team (PPCT) for community care between 01.01.2013–15.09.2016. In Germany, according to a law established in 2007, every child with a life-limiting condition has the right to receive specialized paediatric palliative care. The PPCT is composed of paediatricians, nursing staff and a social worker with additional training in PPC. Home visits are conducted at referral to palliative care and thereafter on a regular basis. The PPCT provides a 24 h/7 days on-call duty by a physician and nursing staff. Besides children and adolescents, who are diagnosed with any LLC, young adults are also eligible for care if the LLC was diagnosed during childhood and adolescence and is unfamiliar to adult palliative care teams [8].

All infants, children, adolescents and young adults (subsequently referred to as “children”) with a diagnosis of a metabolic disease were included. Duration of palliative care was defined as time between the start of palliative home care and interruption of care, death, and the study end (15.09.2016), respectively. Duration of care of those patients, who were already in care at the study start date was counted from the actual start of care and not the study start date (this was also true for the variable 'age at referral/entry into palliative care'). To address that our data and thus duration of care were right-censored (due to study end), we used the Kaplan-Meier method to calculate median duration of care, which was determined as time, at which 50% of the population were still in care.

Patient data were routinely entered into a web interface database by the members of the PPCT and from this extracted and further processed. Information included demographic data, contacts, symptoms, medications, reasons for referral to PPCT, and information on advance care decision-making.

To estimate the symptom burden at referral to palliative care, the median number of symptoms was calculated by counting the number of symptoms documented at each home visit within the first 30 days of care. Patients who were referred to PPC before the start of the electronic documentation and those who were cared for less than 30 days were excluded from the symptom-related analysis only (symptoms in the first/last 30 days of care, respectively). Evaluation of symptoms was performed by the PPC team. For reasons of clarity and better comparability, symptoms were classified into seven categories (general condition, respiratory, neurological, gastrointestinal, urinary tract, body temperature instability, emotional instability/agitation). Four categories (general condition, respiratory, neurological, gastrointestinal) were analysed in more detail regarding symptom frequency. For the symptom severity analysis, all symptoms were analysed. Most individual symptoms were recorded on a severity scale (according to WHO criteria), only the minority was recorded in a binary fashion (cachexia, concentration difficulty, concern, elevated intracranial pressure, fatigue, fear, insomnia, sadness, seizures, tension, weakness).

Frequency data between the groups were compared applying Fisher’s exact test (no adjustment for multiple testing). Further, we conducted significance tests on count and score distributions applying the Wilcoxon rank sum test with continuity correction. All statistical analyses were performed using R (R Core Team 2015).

Symptom scores were calculated from a scale by the formula S = ∑^n^_*i* = 1_ si, where SS is the symptom score, nn the number of observations within a period of 30 or 7 days, ss the scale value with s∈{0,1,2,3,4} for the WHO scale otherwise s∈{0,1,2,3} corresponding to none, light, medium, strong.

All available medications were classified into 26 drug categories, of which only those drug categories, of which at least one drug was prescribed to at least one child, are shown. Due to database programming, we could only analyse the last database entry (corresponding to the prescribed medications at either death/end of study/interruption of care).

The study was approved by the local ethics committee (study number 4969).

## Results

### Patients with metabolic diseases account for 15% of all patients in PPC

In the study period, 198 children were cared for by the PPCT; 29 (14.6%) children were diagnosed with a metabolic disease (Table 1).

**Table 1:** Overview of diagnoses of children with metabolic diseases (*n* = 29)

Lysosomal storage diseases	
Sphingolipidoses	
• Canavan disease	1
• Krabbe disease	1
• Other	2
Sulfatidoses	
• Metachromatic leukodystrophy	3
Gangliosidoses	
• GM2 Gangliosidosis (Tay-Sachs disease)	1
Alexander disease	2
Mucopolysaccharidoses	2
Neuronal ceroid lipofuscinosis	2
Fatty acid disorders	
• Medium-chain acyl-CoA dehydrogenase (MCAD) deficiency	^*^1
Glycine metabolism disorders	
• Hyperglycinemia, nonketotic	2
Mitochondrial diseases	
• Pearson syndrome	^*^2
• Leigh syndrome	^*^2
• *EARS2* mutation	^*^1
• *TMEM70* mutation	^*^1
• etiology unknown	^*^4
Methionine metabolism disorders	
• Sulfite oxidase deficiency	^*^1
Cofactor deficiency disorders	
• Molybdenum cofactor deficiency	^*^1

Those diseases, which can present with metabolic crises are marked with an asterisk

Twelve (41.4%) children were transferred to PPC from hospital, 7 (24.1%) from neurological outpatient services, 4 (13.8%) from home care and 6 (20.7%) from other sources.

Thirteen (44.8%) children were male, median age at referral was 2.6 years, median duration of care was 352 days (317.5 when only counting those patients, who had died during palliative care or whose care had been interrupted), and median number of home visits was 13 (Table 2). Total number of days in care of all patients was 17,821 with a total of 550 home visits; thus, patients were on average seen once every 32 days. Children, who died during palliative care were seen once every 25 days, those children, who were still alive at the end of study were seen once every 40 days. Care was interrupted in 7 (24.1%) patients. In five cases, this was due to a notable stabilization of the clinical situation, in one case the insurance company denied covering the costs and one child was transferred to her home country.

**Table 2:** Demographic data of children with metabolic diseases (*n* = 29)

	Children with metabolic diseases
No.	29
Gender, male (%)	13 (44.8%)
Age at referral, median (range in years)	2.6 (0–24.1)
Age at death, median (range in years)	3.4 (0.1–26.7)
Duration of palliative care, median (range in days)	352 (3–2248)
Home visits, median (range in no.)	13 (1–80)
Deceased children	13 (44.8%)
Place of death, n (%)	
At home	4 (30.8%)
In hospice	1 (7.7%)
In hospital (not ICU)	1 (7.7%)
ICU	7 (53.8%)

Most patients are still alive (16; 55.2%), the majority died in hospital (8; 61.5%), a substantial proportion of those eight children died on ICU (7, 62.5%). When comparing all those children, who later died and were in care fewer than the median of 352 days, they were younger (mean 1.12 years) than those who were in care longer than 352 days (mean 10.4 years).

### Symptom burden is high in children with metabolic disorders at referral and remains high throughout care

**Fig. 1:**
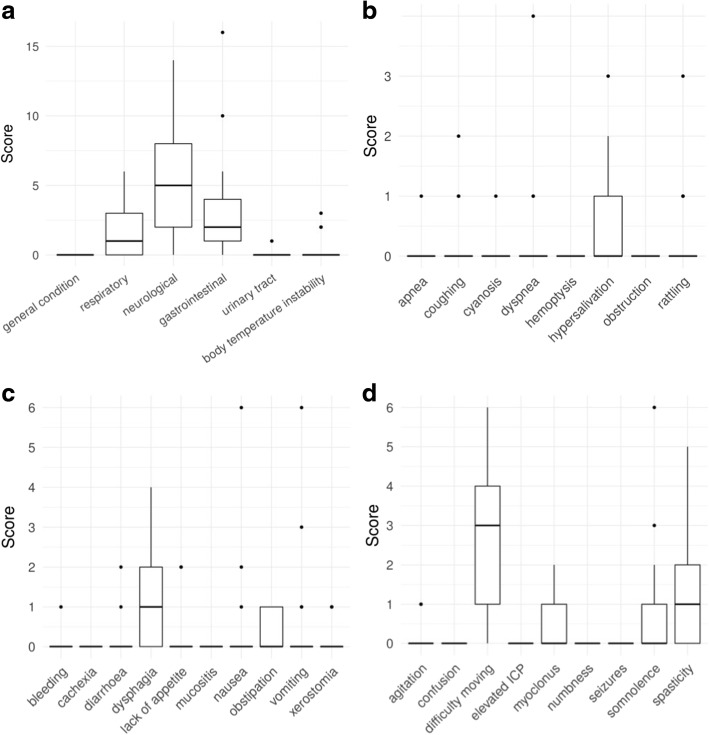
Signs and symptoms of children with metabolic diseases at referral (first 30 days of care). Shown values represent symptom scores as the number of home visits in the first 30 days of care is accounted for. **a**) Overall signs and symptoms. **b**) Detailed respiratory symptoms. **c**) Detailed gastrointestinal symptoms. D) Detailed neurological symptoms

In the 24 children suffering from metabolic diseases for whom we were able to analyse data on the first 30 days of care (for 5 children this time point only was not available), the median number of recorded complaints was 5 (range 1–13) in the first and 6 (range 0–21) in the last 30 days of care. As expected, gastrointestinal, respiratory and neurologic symptoms were predominant, with difficulty moving, seizures, spasticity and somnolence being the most commonly recorded (Fig. 1).

**Fig. 2:**
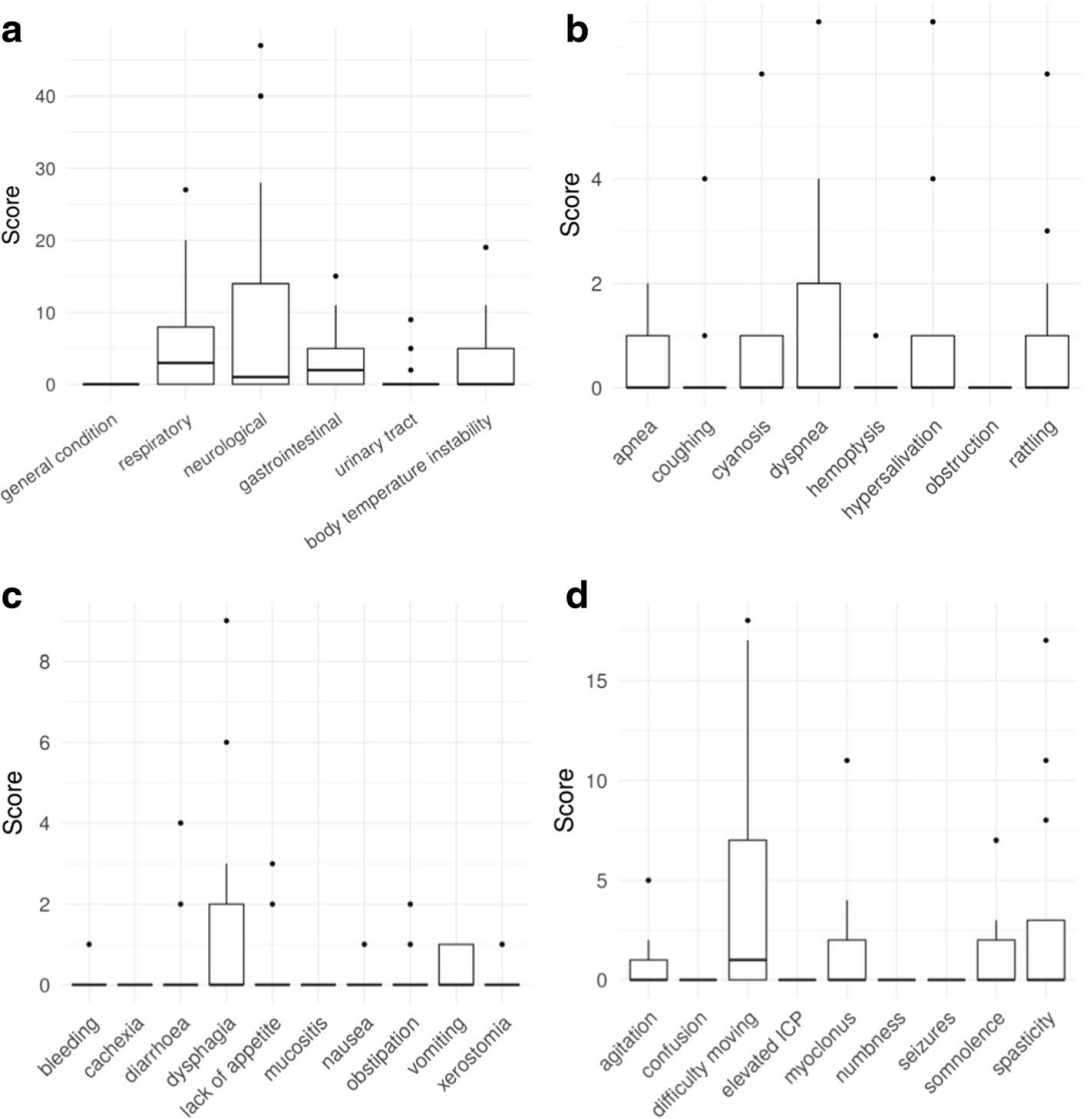
Signs and symptoms of deceased children with metabolic diseases at the end of care (last 30 days of care). Shown values represent symptom scores as the number of home visits in the last 30 days of care is accounted for. **a**) Overall signs and symptoms. **b**) Detailed respiratory symptoms. **c**) Detailed gastrointestinal symptoms. **d**) Detailed neurological symptoms

At least at one home visit within the first 30 days of care, 12 symptoms were recorded to be severe (WHO scale ≥2). Out of the four symptoms, which were recorded to be severe in ≥2 patients, three were neurological (difficulty moving, somnolence, spasticity). At least on one home visit within the last 30 days of care (Fig. 2), 15 symptoms were recorded to be severe (WHO scale ≥2) (9 overlapped between the first and the last 30 days of care). A total of seven symptoms were recorded to be severe in ≥2 patients, which included all four, which were found within the first 30 days of care. With the exception of only two symptoms (hypothermia, nausea), none of the other symptoms ranked as severe decreased in intensity, thus, pointing towards an intractable physical suffering of these children.

As signs of irreversible CNS decline, many children presented with impaired sleep (17.2%), intermittent self-resolving episodes of apnoea (13.8%), slowing of intestinal motility (17.2%), increasing vomiting (24.1%) and agitation (27.6%), muscle spasms (39.9%), and difficulty moving (86.2%) in the last 30 days of care. Other frequently reported symptoms included paleness (48.3%), rattling (34.4%), seizures (41.3%), somnolence (27.6%), spasticity (55.2%), weakness (24.1%), fatigue (24.1%), and hypersalivation (51.7%) (all analysed symptoms refer to the situation in the first 30 days of care).

Three children had a tracheostomy tube, one child was continuously ventilated. However, many other children also presented with an impaired respiratory function, leading to a high frequency of respiratory illness and hospitalizations. Overall, 12 patients were readmitted to hospital (unplanned) at least once (range 1–7), with pneumonias, intractable seizures and feeding tube complications as the most common causes.

### Comparing care between subgroups of patients

Next, we compared children, whose clinical course is potentially characterized by intercurrent metabolic crises (marked by an asterisk in Table 1, *n* = 13, 44.8%) to those, who do not develop crises. There was no difference regarding age at referral or death, duration of palliative care, number of home visits, number of deceased children or places of death. Moreover, signs and symptoms (both in the first and the last 30 days of care) (Additional file 1: Figure S1, Additional file 2: Figure S2) as well as care tools did not significantly differ (Additional file 3: Figure S3). Regarding prescribed medications, most medication categories were similarly employed in both groups, with the only exceptions (all higher in the children without metabolic crises): analgesics (without morphine), antispasmodics (including baclofen) and laxatives (Additional file 3: Figure S3).

Then, we compared those children, who were referred to palliative care early (i.e. less than 1 year of age, *n* = 10) to those, who were referred late (i.e. older than 10 years of age, *n* = 11). The young children had a shorter period of care (median 160 vs. 828 days, *p* = 0.02) resulting in fewer home visits (median 6 vs. 19; *p* = 0.04) and died younger (median age at death 0.7 vs. 18.4 years, *p* = 0.03).

When looking specifically at those patients, who died within the first 30 days after admission to palliative care (*n* = 3), it was noticeable, that -despite low numbers- they were relatively young (mean age 0.7 years, range 0–1.8 years). One each died at home, in hospice and on ICU. Symptom scores were slightly higher in the category respiratory symptoms (not statistically significant) and equal in the category neurological symptoms. Only few medications were prescribed to them, most frequently antiepileptics

### Limiting life-extending measures is difficult for parents of children with metabolic diseases

Discussions about advance care planning (ACP) were conducted at least once. In the following, we analysed the wishes of those parents, whose children died during the course of palliative care or who were still in PPC at the end of the study period (*n* = 22).

More than a third of all parents (11; 37.8%) initially opted for the full range of life-extending measures. Later, two parents changed to a do not attempt resuscitation order. Only four parents (13.8%) already initially decided on a do not attempt resuscitation order. Of those 14 parents, who only wished for some life-support measures, most asked for any type of ventilation support (bag mask or intubation; 7 in total, 31.8%) or antibiotics (also 7, 31.8%). Thirteen children with metabolic disorders died, four at home, one in hospice and eight in hospital (7 in an ICU setting). None of the full code patients died at home.

## Discussion

A substantial proportion of children (14.6%) in PPC presents with inborn metabolic diseases. Symptom burden of these children is high, with gastrointestinal, respiratory and neurologic symptoms being predominant and most of those being intractable. We could not identify statistically significant differences in symptom burden and supportive needs between children with and without metabolic crises. Noteworthy, a high number of children with metabolic diseases died on the ICU.

Most of the children were referred to PPC at a young age (median 2.6 years) due to disease-associated progressive symptoms. Children referred to PPC at less than 1 year of age had a significantly shorter period of care and died earlier/younger pointing towards a rapid disease progression and, thus, the necessity of a very timely identification of and particular attention to the palliative needs of the youngest. In contrast, the median reported age of all children referred to PPC is higher, thus, reflecting the high numbers of children in PPC with conditions with possible life prolonging treatment available and irreversible non-progressive conditions [2, 7, 9]. Indeed, most of these children are referred to PPC due to acute health exacerbations such as infectious complications instead of progressive symptoms.

Most of the children presented with neurologic symptoms. This has previously been reported to be the most prevalent symptom (up to 75%) in all children in PPC [2, 10, 11]. Particularly difficulty moving, seizures, spasticity, and somnolence were not only recorded to be the most frequent but likewise severe and often intractable and, thus, persisting or even increasing during PPC. These symptoms are significant challenges for families and care professionals but very difficult to manage [7, 9, 12]. Thus, further research is urgently needed to improve management of these symptoms.

In accordance with recent reports on children in PPC, also respiratory and gastrointestinal symptoms were frequently present in children with inborn metabolic diseases [9, 12–14]. As one might hypothesize that these are signs of irreversible decline in the CNS including disordered central breathing, one would speculate that these children are more frequently and/or severe affected. However, those numbers differed only slightly from previous reports on children with neurological impairment in PPC [9, 14]. Of note, a remarkable number of children with metabolic diseases recurrently presented with vomiting and feeding difficulties. This is worth paying attention as this might trigger metabolic crises and rapid health decline respectively.

Most children with metabolic diseases reported by us who died during PPC did so in hospital or even in ICU. This is in contrast to previous reports on children dying in PPC, in that most of the children died at home [2, 9, 14, 15]. Looking into these children in more detail, on the one side this is due to the high number of parents who opt for the full range of life-sustaining measure [16]. Metabolic diseases are a fairly complicated diagnosis for parents and non-specialist health care providers to understand. While parents become better at caring for chronically ill children over time, often becoming experts in their child’s care, rapidly progressing conditions may not allow for either acceptance, or development of these skills. In addition, even for the members of the PPCT, the disease trajectory may – to some extent – remain unpredictable and, thus, cause uncertainty about prognosis and medically indicated interventions right up to the best interest of the child. Thus, ACP discussions including the decision whether to stop or maintain life-sustaining measures have been performed with a more open outcome and less restrictive treatment recommendations given to the families, which may partially account for the high number of children dying in hospital or even on ICU [16].

On the other side, some of these children died in the context of interventions such as implantation of a percutaneous endoscopic gastrostomy tube or fixation of a fracture, which might have been caused by metabolic crises triggered by these interventions. Additionally, this might also be a cultural phenomenon, as a high percentage of children with metabolic diseases were of non German origin. A recent study showed large cross-national variations in place of death [17].

Hence, discussions on life-extending measures and the preferred place of death are of particular importance in these children and need more attention in the future. Additionally, as our report represents a single-centre analysis with limited patient numbers, larger multi-centre studies are needed to fully identify and address paediatric palliative care needs of this special patient group.

## Conclusion

About 15% of children with LLC in PPC present with metabolic diseases. Symptom burden is high with neurologic, respiratory and gastrointestinal symptoms being the most frequent and most of those being difficult to treat. In these children, particular attention needs to be addressed to advance care planning.

## Additional files

Additional file 1: **Figure S1.** Signs and symptoms of children with intercurrent metabolic crises (black) and children without metabolic crises (grey) at referral (first 30 days of care). *P*-values obtained from Wilcoxon rank sum tests with continuity correction are show on top. A) Comparison of overall signs and symptoms. B) Comparison of detailed respiratory symptoms. C) Comparison of detailed gastrointestinal symptoms. D) Comparison of detailed neurological symptoms. (JPG 135 kb)

Additional file 2: **Figure S2.** Comparison of signs and symptoms of children with intercurrent metabolic crises (black) and children without metabolic crises (grey) at the end of care (last 30 days). P-values obtained from Wilcoxon rank sum tests with continuity correction are show on top. A) Comparison of overall signs and symptoms. B) Comparison of detailed respiratory symptoms. C) Comparison of detailed gastrointestinal symptoms. D) Comparison of detailed neurological symptoms. (JPG 159 kb)

Additional file 3: **Figure S3.** Overview of the different medication (A) and care tool (B) categories. Children with intercurrent metabolic crises are shown in black and children without metabolic crises in grey. To enable the comparison, relative prescription frequencies are reported. (JPG 252 kb)
