# Supplementary figures and images for: Challenges of palliative care in children with inborn metabolic diseases
Source: Orphanet J Rare Dis. 2018 Jul 9;13:112. doi: 10.1186/s13023-018-0868-5 (PMC6038293; doi:10.1186/s13023-018-0868-5)

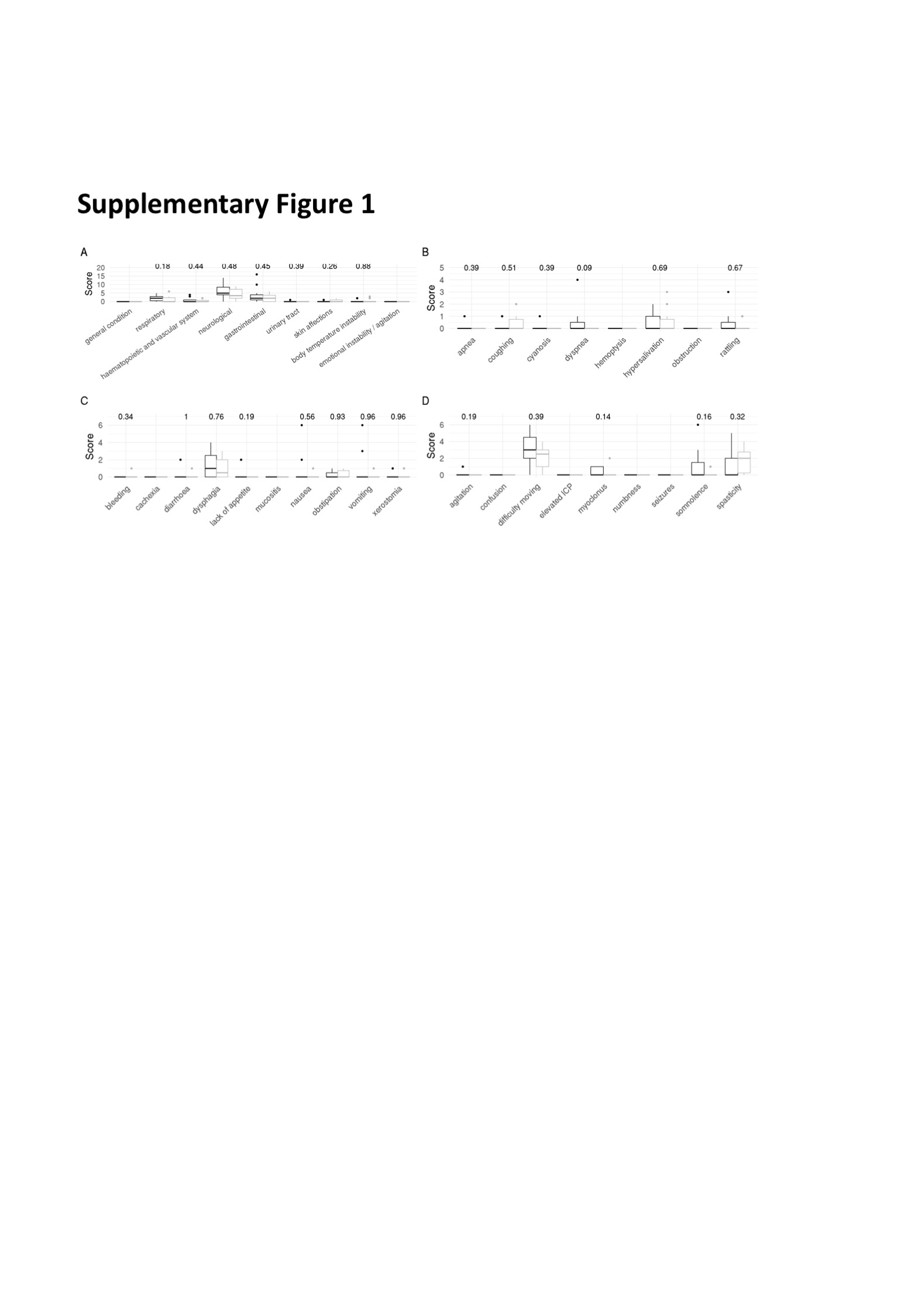

Supplement: Supplementary file 1 — Figure S1. Signs and symptoms of children with intercurrent metabolic crises (black) and children without metabolic crises (grey) at referral (first 30 days of care). P-values obtained from Wilcoxon rank sum tests with continuity correction are show on top. A) Comparison of overall signs and symptoms. B) Comparison of detailed respiratory symptoms. C) Comparison of detailed gastrointestinal symptoms. D) Comparison of detailed neurological symptoms. (JPG 135 kb) [file 13023_2018_868_MOESM1_ESM.jpg]

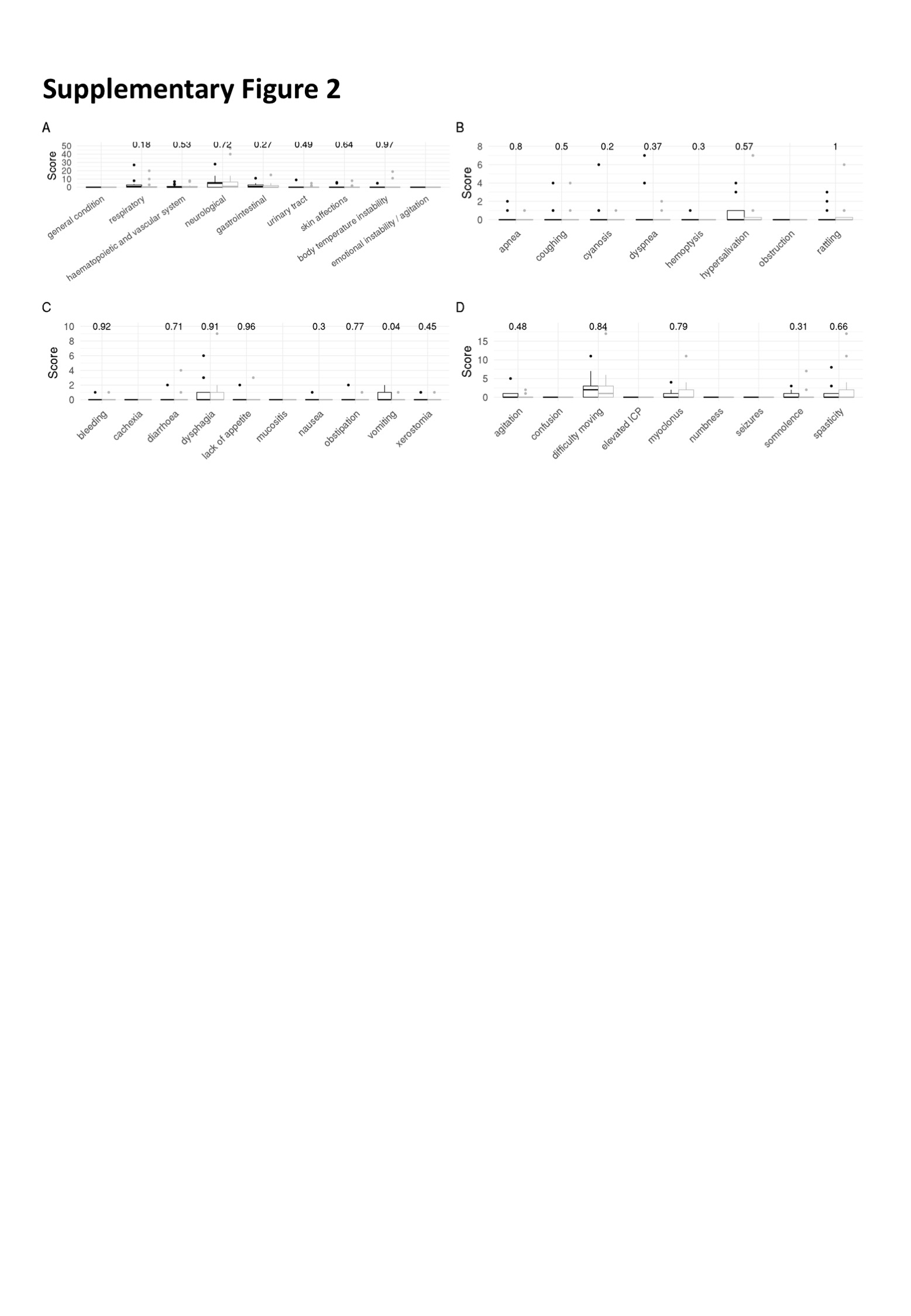

Supplement: Supplementary file 2 — Figure S2. Comparison of signs and symptoms of children with intercurrent metabolic crises (black) and children without metabolic crises (grey) at the end of care (last 30 days). P-values obtained from Wilcoxon rank sum tests with continuity correction are show on top. A) Comparison of overall signs and symptoms. B) Comparison of detailed respiratory symptoms. C) Comparison of detailed gastrointestinal symptoms. D) Comparison of detailed neurological symptoms. (JPG 159 kb) [file 13023_2018_868_MOESM2_ESM.jpg]

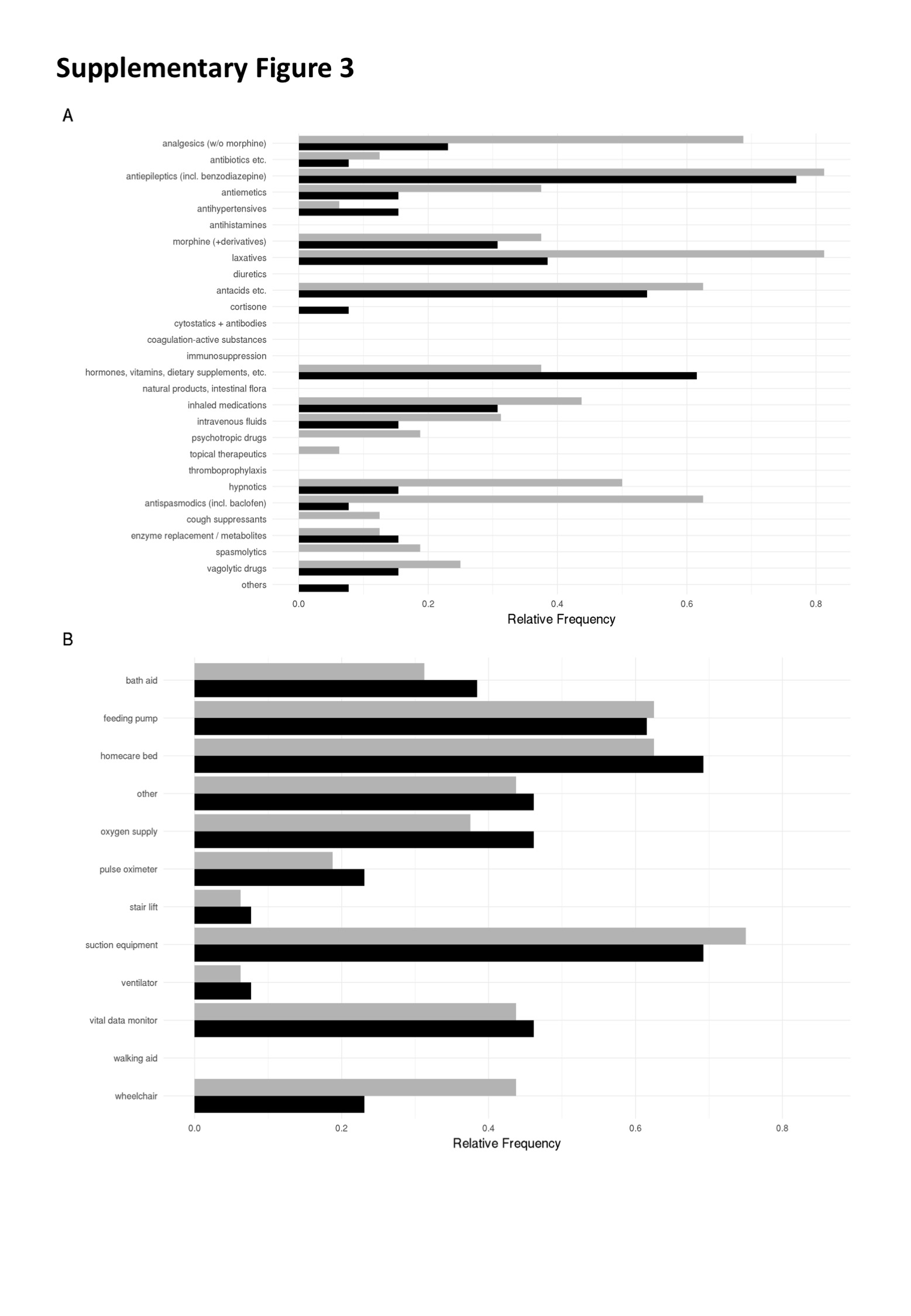

Supplement: Supplementary file 3 — Figure S3. Overview of the different medication (A) and care tool (B) categories. Children with intercurrent metabolic crises are shown in black and children without metabolic crises in grey. To enable the comparison, relative prescription frequencies are reported. (JPG 252 kb) [file 13023_2018_868_MOESM3_ESM.jpg]
